# Recent progress towards understanding the role of DNA methylation in human placental development

**DOI:** 10.1530/REP-16-0014

**Published:** 2016-07-01

**Authors:** Tina Bianco-Miotto, Benjamin T Mayne, Sam Buckberry, James Breen, Carlos M Rodriguez Lopez, Claire T Roberts

**Affiliations:** 1 School of Agriculture, Food and Wine University of Adelaide, Adelaide, South Australia, Australia; 2 Robinson Research Institute University of Adelaide, Adelaide, South Australia, Australia; 3 School of Medicine University of Adelaide, Adelaide, South Australia, Australia; 4 Harry Perkins Institute of Medical Research The University of Western Australia, Crawley, Western Australia, Australia; 5 Plant Energy Biology ARC Centre of Excellence, The University of Western Australia, Crawley, Western Australia, Australia; 6 Bioinformatics Hub University of Adelaide, Adelaide, South Australia, Australia

## Abstract

Epigenetic modifications, and particularly DNA methylation, have been studied in many tissues, both healthy and diseased, and across numerous developmental stages. The placenta is the only organ that has a transient life of 9 months and undergoes rapid growth and dynamic structural and functional changes across gestation. Additionally, the placenta is unique because although developing within the mother, its genome is identical to that of the foetus. Given these distinctive characteristics, it is not surprising that the epigenetic landscape affecting placental gene expression may be different to that in other healthy tissues. However, the role of epigenetic modifications, and particularly DNA methylation, in placental development remains largely unknown. Of particular interest is the fact that the placenta is the most hypomethylated human tissue and is characterized by the presence of large partially methylated domains (PMDs) containing silenced genes. Moreover, how and why the placenta is hypomethylated and what role DNA methylation plays in regulating placental gene expression across gestation are poorly understood. We review genome-wide DNA methylation studies in the human placenta and highlight that the different cell types that make up the placenta have very different DNA methylation profiles. Summarizing studies on DNA methylation in the placenta and its relationship with pregnancy complications are difficult due to the limited number of studies available for comparison. To understand the key steps in placental development and hence what may be perturbed in pregnancy complications requires large-scale genome-wide DNA methylation studies coupled with transcriptome analyses.

## Introduction

Besides mediating maternal–foetal exchange throughout gestation, the placenta plays a major role in orchestrating maternal adaptation to pregnancy by secreting a variety of steroid and peptide hormones. These placental hormones stimulate maternal physiological changes that are essential for pregnancy success. The placenta is unique in several ways. First, although the placenta is a shared organ between mother and foetus, it is an extra-embryonic tissue and is therefore primarily regulated by the foetal genome. Secondly, the placenta separates from mother and foetus after birth, making it a truly transient organ. For these reasons, the epigenetic mechanisms involved in placental development and regulation of gene expression within this tissue may not be subject to the same lifetime epigenetic constraints as other organs that must function throughout an individual’s life.

In humans, from implantation of the blastocyst, the placenta invades the decidua colonizing and transforming the uterine spiral arterioles to sequester a maternal blood supply for efficient maternal–foetal exchange (Roberts 2010). Invading placental extravillous cytotrophoblasts employ molecular mechanisms that closely match those of a metastatic tumour (Murray & Lessey 1999); however, although this process is strictly controlled both spatially and temporally in the placenta, it is somewhat dysregulated in cancer. Such mechanisms are not fully understood but include complex interactions between both extravillous cytotrophoblasts and maternal endothelium and leucocytes (Graham & Lala 1992, Lyall 2002, Yu* et al*. 2015). Emerging evidence suggests that epigenetic regulation of the placental transcriptome is important for the molecular control of placental growth and differentiation. This review highlights some of the complexities of placental DNA methylation in humans and how this process may be disrupted in some pregnancy pathologies. The focus of this review is genome-wide DNA methylation studies on human placental tissues, what we have learnt from these studies and what remains to be discovered.

## Epigenetics

Epigenetics is often defined as modifications that affect genome architecture and accessibility which can influence gene transcription, without altering the underlying DNA sequence. Such modifications include DNA methylation and histone modifications that, unlike changes to the DNA sequence, may be reversible. This review focuses on the most widely studied epigenetic modification: DNA methylation, which is the addition of a methyl group (–CH_3_) to cytosine bases, is a process catalyzed by DNA methyltransferases (*DNMT1*, *DNMT3A* and *DNMT3B*). *DNMT1* maintains and repairs established DNA methylation, whereas *DNMT3A* and *DNMT3B* are involved in *de novo* DNA methylation (Goll & Bestor 2005, Denis* et al*. 2011). Typically, DNA methylation of gene regulatory regions is associated with repression of gene expression; however, many genome-wide DNA methylation studies have demonstrated that this is not always the case (Ke* et al*. 2010, Moarii* et al*. 2015, Becket* et al*. 2016). DNA methylation is important in genomic imprinting and X chromosome inactivation in females. It has been widely studied in diseases and has been used as a biomarker for predicting disease or environmental exposures (Portela & Esteller 2010).

## DNA methylation in the placenta

DNA methylation plays a crucial role during cellular differentiation and development (Ji* et al*. 2010, Kim* et al*. 2010). Studying DNA methylation in the placenta is complicated by the presence of several different cell types. The majority of studies investigating this topic have used chorionic villi, which have a different DNA methylation profile than embryonic tissues, the maternal decidua or foetal membranes (amnion and chorion) (see Robinson & Price 2015). Chorionic villi are the site of maternal–foetal exchange and hormone production and contain a mixture of cell types with cells derived from both the trophectoderm and the inner cell mass. The cellular composition of individual placentas varies and this is most apparent in the presence of a pregnancy complication (Mayhew* et al*. 2004).

## Hypomethylation of placental tissue

It has been known for some time that the genome of the placenta is hypomethylated compared with that in other healthy tissues (Ehrlich* et al*. 1982, Fuke* et al*. 2004). However, how or why the placental genome is hypomethylated remains unclear, but may reflect the heterogeneous nature of placental tissue and the corresponding different DNA methylation profiles of distinct cell populations. Shortly after fertilization, the embryonic DNA becomes largely demethylated (Smith* et al*. 2012). In the following days, the cells in the inner cell mass rapidly undergo *de novo* DNA methylation but the trophectoderm remains hypomethylated (reviewed in Robinson & Price 2015). However, this information has been obtained in the extensively and easily studied mouse model. Dissecting out the DNA methylation profiles in different cell types across development in human embryos is technically more difficult than in mice. Some data suggest that DNA methylation throughout embryo development in humans differs when compared with mice (Guo* et al*. 2014). Furthermore, it appears that the DNA methylation levels in the trophectoderm are marginally lower than those in the inner cell mass in humans, but these findings are based on observations in very few samples (Guo* et al*. 2014).

The chorionic villi of the placenta are composed of cells derived from both the trophectoderm (all populations of trophoblasts) and the inner cell mass (extra-embryonic mesoderm and endoderm progenitors comprising the villous stroma and blood vessels). The villous syncytiotrophoblast is the major cell type in the placenta and is derived from the trophectoderm via the villous cytotrophoblasts which fuse to form the syncytium (Gude* et al*. 2004). Therefore, hypomethylation of the placental genome may reflect the maintenance of the early hypomethylated state of the trophectoderm through development. It is also known that *DNMT1* expression in the placenta is reduced with mono-allelic DNA methylation of the promoter region (Novakovic* et al*. 2010, Das* et al*. 2013), which may also contribute to the hypomethylated state of the placental methylome.

## Partially methylated domains in the placenta

Hypomethylation within the placenta is not uniform but occurs in large domains (>100kb) called partially methylated domains (PMDs) which are regions of reduced DNA methylation that cover approximately 40% of the placental genome (Schroeder* et al*. 2013). PMDs are unique to a few different tissue types that include the placenta, some cultured cells and cancer (Lister* et al*. 2011, Schroeder & LaSalle 2013, Schroeder* et al*. 2013). Genes within placental PMDs are typically repressed, have tissue-specific functions and their methylation status is maintained throughout gestation (Schroeder* et al*. 2013). However, even though PMDs seem to be a characteristic trait of the placental methylome, the majority of studies published to date have largely ignored them; therefore, it remains unclear why the placental methylome is characterized by PMDs. It is also yet to be determined when PMDs are first established in the trophoblast or placenta, what roles PMDs and the genes located within them play and whether they are disrupted in pregnancy complications.

## Foetal sex differences in DNA methylation

Although the placental genome contains fewer methylated cytosines than other tissues (Ehrlich* et al*. 1982, Fuke* et al*. 2004), a large study in 248 placentas has shown that there is a large range of DNA methylation of 2–5% (Dwi Putra* et al*. 2014), but the reason for this variation requires further investigation. The regions with reduced DNA methylation included long interspersed nuclear elements (LINE1) and Alu repeats, as well as CpG island promoters associated with X-linked genes (Cotton* et al*. 2009). By using an Illumina DNA methylation microarray, Cotton *et al.* (2009) assessed 84 sites within X chromosome-associated promoter CpG islands and found that overall DNA methylation of these sites was reduced in female placentas to a greater extent than in male placentas, suggesting that there was DNA methylation loss at the inactive X chromosome. This was further supported by pyrosequencing assays for CpG island-associated promoters on the X chromosome (Cotton *et al*. 2009).

**Figure 1:**
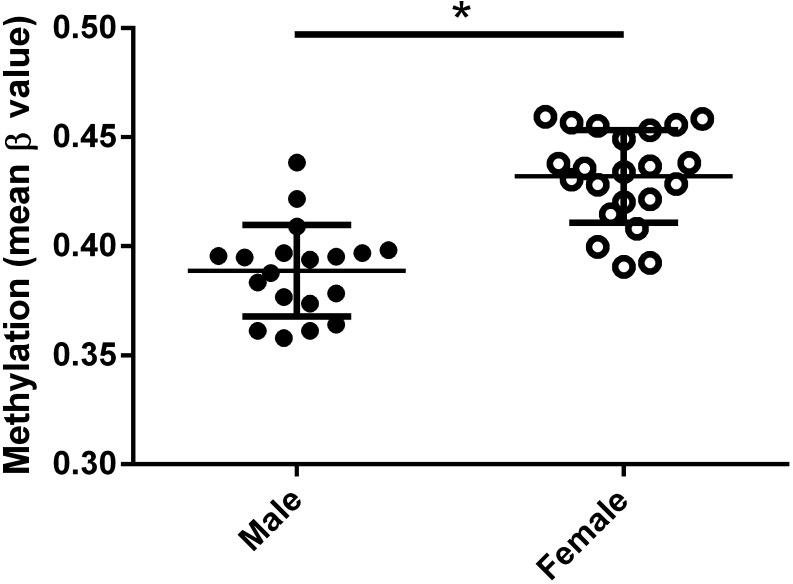
DNA methylation is higher on the X chromosome in placentas from female bearing pregnancies. DNA methylation levels were assessed at 8346 CpG sites on the X chromosome. DNA methylation of placental tissue from three publicly available data sets (GSE44667, GSE54399 and GSE57767) for a total of 19 male and 22 female term uncomplicated placentas was assessed. Probes that had missing values for samples were discarded, leaving 8346 X chromosome probes for all samples. Normalization was performed using the beta-mixture quantile normalization (BMIQ) method, which corrects for the two different designs of probes, followed by quantile normalization (Teschendorff* et al*. 2013). Batch effects were corrected using the Combat function implemented in the ChAMP Bioconductor package (Leek* et al*. 2012, Morris* et al*. 2014). Sample sex was identified using the minfi package in which the median value of theβvalues for probes that mapped uniquely for the X and Y chromosome, respectively, were first determined (Aryee* et al*. 2014). This resulted in the identification of 19 male and 22 female placentas. The overall DNA methylation for the X chromosome in each sample was calculated by taking the overall meanβvalue of all the probes that map to the X chromosome.

Our comparison of DNA methylation across 8346 CpG sites on the X chromosome using three datasets of placental tissue from uncomplicated term pregnancies (GSE44667, GSE54399 and GSE57767) (Blair* et al*. 2013, Anton* et al*. 2014) including 22 female and 19 male placentas indicated that the X chromosome from female placentas was more methylated than the X chromosome from male placentas (unpaired *t*-test, mean (female)=0.432; mean (male)=0.389, *P*-value=9.552e-08) (Fig. 1). When only the CpG sites within the 5′-UTR were analysed (2512 probes), DNA methylation on the X chromosome was higher in females than in males, as expected and seen for other organs (Yasukochi* et al*. 2010, Cotton* et al*. 2011, Hall* et al*. 2014, Joo* et al*. 2014).

## Cell-type differences in DNA methylation

As previously mentioned, the placenta is composed of several different cell types (Kanellopoulos-Langevin* et al*. 2003) that are likely to have their own unique corresponding methylome. Grigoriu *et al*. (2011) compared DNA from whole placenta with corresponding isolated fibroblasts and cytotrophoblasts separated from the same tissues using an enzymatic and magnetic bead separation methodology. By comparing DNA methylation profiles using the Illumina Infinium Human Methylation 27K BeadChip Array, the authors were able to identify 61 probes for genomic regions that were differentially methylated between whole placenta and cytotrophoblasts, 315 between whole placenta and fibroblasts and 442 between fibroblasts and cytotrophoblasts (Grigoriu* et al*. 2011). Interestingly, the cytotrophoblast DNA methylation profiles clustered with the whole placenta samples and the fibroblasts clustered on their own (Grigoriu* et al*. 2011). These results indicate that the methylome differs between the different cell types that make up the placenta and that this should be considered when performing DNA methylation studies in this organ. This is supported by other studies that have shown differential DNA methylation changes in distinct placental cell populations (Novakovic* et al*. 2008, 2011*a*).

Besides 5-methylcytosine, there are other derivatives, such as 5-hydroxymethylcytosine, that arise from the oxidation of 5-methylcytosine in the process of DNA demethylation (Hernando-Herraez* et al*. 2015). The precise role and function of 5-hydroxymethylcytosine require further study; however, it is important in development and disease (Tan & Shi 2012). In a recent study by Fogarty *et al*. (2015), differences in the levels of 5-methylcytosine and 5-hydroxymethylcytosine between syncytiotrophoblast and cytotrophoblasts were assessed using immunohistochemical quantification (Fogarty* et al*. 2015). This study showed higher levels of 5-methylcytsoine in cytotrophoblasts, whereas 5-hydroxymethylcytosine was more abundant in syncytiotrophoblast (Fogarty* et al*. 2015). The biological reason behind this difference is unclear but suggests that the methylomes may vary substantially between the different cell types that make up the placenta, which may be consistent with the differentiation state of the two villous trophoblast populations. The extent to which these epigenetic differences influence and define different cell populations in the placenta remains to be elucidated.

## DNA methylation changes across gestation

One of the first studies to characterize DNA methylation across gestation used HPLC to show that global DNA methylation levels increase with gestational age (Fuke* et al*. 2004), which has been subsequently confirmed by others using different methodologies (Novakovic* et al*. 2011*b*, Price* et al*. 2012). Novakovic *et al.* (2011*b*) used Illumina arrays to show that DNA methylation increases from first trimester to third trimester with levels of DNA methylation very similar between second and third trimester. The authors suggest that the change in cell composition and differentiation of cells that occurs as gestation progresses may contribute to these differences, as well as the bias of this methodology with respect to the actual methylation sites assessed by the array probes (Novakovic* et al*. 2011*b*).

## DNA methylation changes and pregnancy complications

The analysis of the placental methylome in pregnancy complications such as pre-eclampsia (PE), intrauterine growth restriction (IUGR), preterm birth (PTB) and gestational diabetes mellitus (GDM) has largely been performed using Illumina Infinium Human Methylation BeadChip Arrays (Banister* et al*. 2011, Lambertini* et al*. 2011, Jia* et al*. 2012, Blair* et al*. 2013, Ruchat* et al*. 2013, Anton* et al*. 2014, Chu* et al*. 2014, Liu* et al*. 2014, Finer* et al*. 2015, Hillman* et al*. 2015, Petropoulos* et al*. 2014) (Table 1). These studies identify differential methylation between placentas from controls and the pregnancy complication under investigation, with some studies showing overlap of differentially methylated sites in umbilical cord blood (Ruchat* et al*. 2013, Finer* et al*. 2015). However, Hillman *et al.* (2015) found no differences in DNA methylation in placenta, but observed differences in cord blood when comparing IUGR with controls. Interestingly, these differences in DNA methylation profiles in cord blood between healthy pregnant women and women diagnosed with pregnancy complications have also been reported in a preliminary study between healthy women and women diagnosed with either PE or PTB using maternal peripheral blood sampled at 15-week gestation, long before their diagnosis (Bianco-Miotto* et al*. 2015).

**Table 1:** Summary of DNA methylation studies in placenta using genome-wide approaches.

**Reference**	**Method**	**Population**	**Sample (*n*)**	**Key findings**
Finer* et al*. (2015)	Illumina HM450	UK (South Asian origin)	Term placenta (25 GDM, 18 controls) Cord blood (27 GDM, 21 controls)	More hypermethylated sites observed in both placenta and cord blood samples with GDM compared with the controls 4219 probes and 13,561 probes were differentially methylated between GDM and controls in placental tissue and cord blood respectively. 378 probes were common in placental tissue and cord blood
Hillman* et al*. (2015)	Illumina HM450	UK	Placenta (23 IUGR, 22 controls) Cord blood (27 IUGR, 18 controls)	No differentially methylated positions were observed in placental tissue between IUGR and controls. 839 differentially methylated regions were revealed in cord blood
Anton* et al*. (2014)	Illumina HM450	USA (80% African American 20% Other)	Placenta (19 term PE, 12 PTB+PE, 14 controls)	229 and 3411 loci were differentially methylated in PE and PTB+PE in comparison with the controls. Validation of four genes that were differentially methylated by qPCR confirmed altered mRNA expression
Chu* et al*. (2014)	Illumina HM27	USA	Term placenta (24 PE, 24 controls)	PE samples were hypomethylated compared with controls. Clustering revealed that foetal sex is associated with DNA methylation irrespective of disease state
Liu* et al*. (2014)	Methylated-CpG island recovery assay (MIRA)	China	Placenta (27 PE, 28 GDM, 30 control)	8191 (2140 genes) and 10,424 (2644 genes) differentially methylated regions were identified in PE and GDM compared with controls respectively 65% of the genes with different methylation revealed concordant changes in methylation between PE and GDM
Ou* et al*. (2014)	Illumina HM450	China	First-trimester placenta and maternal blood (three of each) Term placenta and maternal blood (two of each)	Identified 2944 and 5218 hypermethylated CpG sites that were foetal specific and found an overlap of 2613 differentially methylated sites between maternal blood and placenta tissue (present in both first- and third-trimester samples)
Petropoulos* et al*. (2014)	Illumina HM450	Canada	Term placenta (seven GDM, seven controls)	2021 CpG sites (981 genes) were differentially methylated between GDM and controls
Xiang* et al*. (2014)	MeDIP-Seq and Illumina HM450	China	First-trimester placenta and maternal blood (14 of each)	Using both assays, 3759 CpG sites in 2188 regions were differentially methylated between maternal blood and placenta
Blair* et al*. (2013)	Illumina HM450	Canada	Third-trimester placenta (20 EOPET, 20 controls)	38,840 CpG sites were altered in EOPET vs controls. Gene expression microarray of a subset of samples (eight of each) showed negative correlation of gene expression changes with DNA methylation alterations
Ruchat* et al*. (2013)	Illumina HM450	Canada	Term placenta and cord blood (30 GDM, 14 controls)	3271 and 3758 genes were differentially methylated in controls vs GDM in placenta and cord blood, respectively, 25% common to both placenta and cord blood. The genes that were differentially methylated were involved in metabolic disease
Schroeder* et al*. (2013)	Illumina HM450 and MethylC-Seq & RNA-Seq	USA	Placenta (five first, ten second, 21 third trimester) for 450K Three term placentas for MethylC-Seq	Identified partially methylated domains (PMDs) cover 37% of the placental genome. RNA-seq revealed that genes with PMDs are repressed. 450K data showed that PMDs are conserved throughout gestation
Gordon* et al*. (2012)	Illumina HM27	Australia	Term placenta (eight MZ, seven DZ pairs)	MZ pairs showed greater similarity in intra-pair DNA methylation than DZ pairs
Jia* et al*. (2012)	MeDIP+NimbleGen human CpG island promoter microarray (385K)	China	Pooled term (3) compared with pooled PE (3)Validation (nine control, nine PE)	3280 genes differentially methylated between controls and PE. Six genes (*CAPN2*, *EPHX2*, *ADORA2B*, *SOX7*, *CXCL1*, *CDX1*) were validated by bisulphite sequencing
Turan* et al*. (2012)	Illumina HM27+IlluminaHumanHT-12 v3 Expression BeadChip	USA	Term placenta (48)	Correlated DNA methylation levels with birth weight
Banister* et al*. (2011)	Illumina HM27	USA	Term placenta (89 SGA, 117 controls)	Identified 22 differentially methylated loci that are associated with SGA
Novakovic* et al*. (2011*b*)	Illumina HM27	Australia and Canada	Placenta (18 first, ten second, 14 third trimester)	An increase in overall genome methylation observed from first to third trimester. First-, second- and third-trimester cluster separately on a dendrogram
Lambertini* et al*. (2011)	MeDIP+Affymetrix Human Tiling Array 2.0R	USA	Term placenta (10 control, 7 IUGR)	Identified 113,020 genome-wide differentially methylated regions
Rakyan* et al*. (2008)	MeDIP+custom microarray	UK (European)	Term placenta (3)	Identified tissue-specific differentially methylated regions in the placenta

Illumina HM27, Illumina Infinium Human Methylation 27K BeadChip; Illumina HM450, Illumina Infinium Human Methylation 450K BeadChip; MethylC-Seq, whole genome bisulphite sequencing; MeDIP, methylated DNA immunoprecipitation; DZ, dizygotic twins; MZ, monozygotic twins; EOPET, early-onset pre-eclampsia; GDM, gestational diabetes mellitus; IUGR, intrauterine growth restriction; PE, pre-eclampsia; SGA, small for gestational age.

Comparison between studies is made difficult due to different methodologies used; however, for studies in which comprehensive gene lists were available, we compared the overlap in differentially methylated genes. For GDM compared with uncomplicated pregnancies, we were able to compare two studies (Finer* et al*. 2015, Petropoulos* et al*. 2014) and found 91 genes that overlapped between the two studies. For PE, there appeared to be much less overlap with only two genes (*DAPK3*, *PAPPA2*) in common between the two studies that were compared (Blair* et al*. 2013, Chu* et al*. 2014). This is not surprising as Blair *et al*. (2013) assessed samples from women with early-onset pre-eclampsia and compared them with samples from gestational age-matched controls, whereas Chu *et al*. (2014) assessed samples from women with PE including 15 of 24 from women with term pre-eclampsia and compared them with term controls (Chu* et al*. 2014). Term PE has a very different aetiology to early-onset disease and preterm controls, although matched for gestational age could not be considered to be uncomplicated pregnancies (Andraweera* et al*. 2012). Despite this, Chu *et al*. (2014) found only one gene to be differentially methylated in early-onset versus term PE.

Of particular interest, only one study thus far has examined DNA methylation using methylC-seq (Schroeder* et al*. 2013), but was hampered by a small sample size (just three term placenta samples). MethylC-seq (Schroeder* et al*. 2013) uses sodium bisulphite treatment to convert all non-methylated cytosines to thymine, allowing single-base pair resolution of all 5mC sites within a sample. With sufficient coverage using high-throughput sequencing, the entire 5mC methylome can be identified in one methylC-seq library. This protocol is in stark contrast to the predominant use (driven by cost) of DNA methylation microarrays, which cover only ∼1–2% of the genome (Plongthongkum* et al*. 2014). Consequently, the majority of the placental methylome remains unexplored. This shortcoming is further compounded by the lack of studies quantifying both DNA methylation and gene expression in matched samples, which is crucial in linking altered DNA methylation to changes in gene regulation. There are few studies that have assessed gene expression and DNA methylation in the same samples and both of these only studied the term placenta (Turan* et al*. 2012, Schroeder* et al*. 2013). Without investigating gene expression together with DNA methylation, it is difficult to elucidate the role of DNA methylation in gene regulation in the placenta and how this may be disrupted in pregnancy complications. With reducing costs for next-generation sequencing, we anticipate more genome-wide placental DNA methylation studies in the future, which would increase our knowledge of placental DNA methylation and its effects on gene transcription in health and disease.

## Conclusion

Although there are several studies of genome-wide DNA methylation profiling in the human placenta, our knowledge remains insufficient to draw well-supported conclusions about the role of DNA methylation in placental development, particularly since most studies have very small sample sizes. There are currently very few whole-genome bisulphite sequencing studies that provide comprehensive profiles of placental DNA methylation across gestation, and how these epigenetic modifications correlate with gene expression. What is also unclear is the role of other epigenetic modifications such as 5-hydroxymethylation and histone modifications in placental gene regulation. An integrated analysis of the placental epigenomic landscape may be required to begin elucidating the role of the placental epigenome in normal development and in pregnancy complications. Finally, as recently highlighted (Robinson & Price 2015), without understanding the distinct methylation profiles of the different cell types that make up the placenta, it is difficult to understand the role of DNA methylation in healthy placentas and what changes accompany pathology.

## Declaration of interest

The authors declare that there is no conflict of interest that could be perceived as prejudicing the impartiality of the research reported.

## Funding

C T R is supported by a National Health and Medical Research Council of Australia (NHMRC) Senior Research Fellowship (GNT1020749) and NHMRC project grant (GNT1059120) awarded to CTR and TB-M. SB is supported by a NHMRC and Australian Research Council (ARC) Dementia Research Development Fellowship (GNT1111206).
